# Efficacy of laparoscopic versus open abdominal radical hysterectomy on stress response and urodynamic status in patients with early cervical cancer

**DOI:** 10.12669/pjms.41.12.12669

**Published:** 2025-12

**Authors:** Ping Gong, Haitao Wang, Yuanjing Wang

**Affiliations:** 1 Ping Gong, Department of Gynecology and Obstetrics, Beijing Shijitan Hospital, Capital Medical University, Beijing 100038, P.R. China; 2 Haitao Wang, Department of Urology, Beijing Shijitan Hospital, Capital Medical University, Beijing 100038, P.R. China; 3 Yuanjing Wang, Department of Gynecology and Obstetrics, Beijing Obstetrics and Gynecology Hospital, Capital Medical University, Beijing 100026, P.R. China

**Keywords:** Abdominal radical hysterectomy, Early cervical cancer, Laparoscopic radical hysterectomy, Stress response, Urodynamic status

## Abstract

**Objective:**

To compare the impact of open abdominal radical hysterectomy (ARH) and laparoscopic radical hysterectomy (LRH) on stress response and urodynamic status of early cervical cancer (CC) patients.

**Methodology:**

Clinical data of 115 patients with early-stage cervical cancer (CC) who underwent surgery at Beijing Shijitan Hospital, Capital Medical University, between December 2021 and June 2024 were retrospectively analyzed. Based on the surgical approach, patients were divided into the ARH group (n = 59) and the LRH group (n = 56). Perioperative status, stress response, and urodynamic indicators before and after the surgery, and the incidence of complications were compared between the two groups.

**Results:**

The perioperative indicators (surgical duration, intraoperative blood loss, anal exhaust duration, number of dissected lymph nodes, and the length of hospital stay) of patients in the LRH group were better than those of the ARH group (*P*<0.05). After the surgery, the levels of serum angiotensin II (Ang II), growth hormone (GH), and cortisol (Cor) increased in both groups compared to preoperative levels and were significantly lower in the LRH group than in the ARH group (*P*<0.05). After the surgery, bladder capacity and maximum urethral pressure (MUP) increased, whereas residual urine volume (RUV) decreased in both groups compared with preoperative levels. Patients who underwent LRH had greater bladder capacity and MUP and lower RUV compared to the ARH group (P < 0.05). The incidence of complications in the LRH group was significantly lower than that in the ARH group (P < 0.05).

**Conclusions:**

Compared with ARH, LRH for early CC provides greater benefits in the early postoperative period, including a lower stress response and better improvement in short-term urodynamic indicators.

## INTRODUCTION

Radical hysterectomy remains the standard surgical treatment for early-stage cervical cancer (CC), and can be performed using either open abdominal surgery or a minimally invasive approach.^1^,^2^ Abdominal radical hysterectomy (ARH), which does not require additional equipment and cooperation from surgical teams, has been established as the standard care for early CC patients, as it is easier to carry out and promote in primary hospitals.^3^,^4^ Additionally, ARH allows easy assessment of the lesions and the lymph nodes, which is crucial for complete resection, more thorough treatment, and better prognosis. 4,5 Compared to ARH, laparoscopic radical hysterectomy (LRH) is more technically challenging but results in smaller incisions and less trauma.^5^ Due to the magnification effect of LRH, this technique provides a clearer field of view and more detailed anatomy. It is associated with lesser damage to normal tissues around the tumor, thus facilitating postoperative recovery.^5^,^6^

Despite differences, both approaches are associated with a certain extent of surgical trauma, which is often accompanied by strong acute pain at the surgical site. The resulting stress response is accompanied by the excessive release of stress hormones such as serum angiotensin II (Ang II), growth hormone (GH), and cortisol (Cor),^7^ and various complications, including urinary retention.^8^,^9^ Lower urinary tract symptoms are the most common complication after radical hysterectomy for CC.^10^ Studies show that urinary retention and other complications in CC patients are likely to occur when bladder contractility is blocked and the detrusor sphincter coordination is out of balance.^11^,^12^

Recent comparative trials and meta-analyses have attempted to compare LRH and ARH in terms of CC recurrence, patient survival, and quality of life, with conflicting results.^13^ This underscores the need for real-world evidence of the perioperative recovery and functional outcomes of CC patients after LHR or ARH, with a special focus on stress response and urodynamic recovery.

This study retrospectively analyzed the treatment records of 115 early CC patients who received ARH or LRH to compare the impact of two surgical approaches on stress response and urodynamic status in the early postoperative period. These findings may suggest surgical decision-making for early-stage CC patients.

## METHODOLOGY

This retrospective cohort study included treatment records of early CC patients who underwent surgical treatment (ARH or LRH) at Beijing Shijitan Hospital, Capital Medical University from December 2021 to June 2024.

### Ethical approval:

The study was approved by the hospital ethics committee Ref: IIT2024-134-002, Date: October 12^th^ 2024. Patients were divided into ARH and LRH groups based on the surgical approach.

### Inclusion criteria:

- Meet the diagnostic criteria for cervical cancer.
- The disease stage Ia - Iia.
- Complete ARH or LRH treatment for the first time.
- Complete clinical data.

### Exclusion criteria:

- Patients with a history of dependence on sedatives or analgesics.
- Patients with other malignant tumors (such as lung cancer, stomach cancer).
- Patients with coagulation dysfunction
- Patients with preoperative disorders in defecation, urination, and autonomous behavior.

### ARH:

Under general anesthesia, the patient was placed in the dorsal lithotomy position. A midline lower abdominal or Pfannenstiel incision was made. A type C radical hysterectomy was performed, including en bloc removal of the uterus, cervix, parametria, and upper vagina, together with bilateral pelvic lymphadenectomy. The uterine vessels and uterosacral and cardinal ligaments were ligated and transected at an appropriate distance from the cervix. After adequate hemostasis and placement of a pelvic drain, the abdominal incision was closed in layers.

### LRH:

Under general anesthesia, the patient was placed in the bladder lithotomy position, head low and feet high. A uterine lifting device was placed in the vagina to evaluate the invasion of cancer cells into the cervix. The pelvic peritoneum was opened, and multiple adipose and lymphatic tissue areas were removed. The external iliac artery and vein vessels were freed to open the vascular sheath, clean hidden lymph nodes, and collect lymph node tissue. Pelvic ligaments were fully exposed. When isolating cervical tissue, high-level electrocoagulation was used, the bladder peritoneum was opened, and the uterus and bladder were pushed downwards. An ultrasonic knife was used to locally free the ureteral tunnel, ensuring it could reach the bladder angle and shear the bladder ligament. Vaginal and cervical tissues were separated, fully exposing the main ligament and completely removing the sacral ligaments on both sides. The annular vaginal wall was dissected layer by layer in the form of unipolar electrocoagulation; a catheter was placed, and the incision was closed.

### Collected indicators:

The primary endpoints of this study were the changes in stress response indicators (Ang II, GH, and Cor) and urodynamic parameters (bladder capacity, MUP, and RUV), which reflect physiological responses to surgical trauma and recovery. The secondary endpoints included perioperative indicators (surgical duration, intraoperative blood loss, time to anal exhaust, number of lymph node dissections, and hospital stay duration) and the incidence of postoperative complications.

### The following data were collected from all patients:

- Basic characteristics, including age, body mass index (BMI), pathological type, International Federation of Gynecology and Obstetrics (FIGO) staging, marital status, etc.
- Perioperative indicators, including surgical duration, intraoperative blood loss, anal exhaust duration, number of lymph node dissections, and length of hospital stay.
- Stress response indicators one day before the surgery and three days after the surgery. Briefly, serum levels of angiotensin II (Ang II), growth hormone (GH), and cortisol (Cor) were measured by enzyme-linked immunosorbent assay.
- Urodynamic status indicators one day before the surgery and three days after the surgery, including bladder capacity, maximum urethral pressure (MUP) and residual urine volume (RUV), were assessed by WEIST8000 urodynamic analyzer. First, a routine free urine flow rate measurement was performed. An F8 double-lumen pressure measuring catheter was inserted through the urethra, and a rectal pressure tube was placed transrectally. Surface electrodes were placed at the anal sphincter and connected to corresponding pressure sensors and water pumps, which were used to inject water at a rate of 50mL/min. Bladder pressure was measured during the filling phase, and each urinary sensation was recorded. After filling the bladder to its maximum capacity, the patient was instructed to collect urine using a collection device. Simultaneous measurement of urinary pressure flow rate and residual urine volume was performed during urination. Electromyographic changes during the bladder filling and urination periods were recorded. All data were processed and stored on the computers.
- The occurrence of complications, including intestinal obstruction, urinary retention, infection, fallopian tube injury, etc.

### Statistical analysis:

The data were input into Microsoft Excel and analyzed using SPSS version 26.0 (IBM Corp, Armonk, NY, USA). According to the Shapiro-Wilk test evaluation of distribution normality, continuous variables were reported as means and standard deviations (SD), and an independent sample t-test was used for inter-group comparisons. An independent sample t-test was used for inter-group comparison, and a paired t-test was used for intra-group comparison before and after the surgery. The non-normally distributed data were represented by a median and interquartile range, and the Mann-Whitney U test was used for inter-group comparison. The count data were represented by the number of cases, using the chi-square test or Fisher’s exact probability method. PRISM8.0 software (GraphPad, San Diego, USA) was used to draw bar charts of preoperative and postoperative stress response and changes in urodynamic indicators for two groups of patients. A p-value less than 0.05 was considered statistically significant. All reported p-values were two-sided.

## RESULTS

A total of 115 patients met the criteria and were included in the analysis. The age of the cohort ranged from 35 to 67 years, with an average age of 49.21 ± 7.46 years. Of 115 patients, 56 underwent LRH and 59 underwent ARH, with no significant difference in the baseline clinical characteristics between the two groups (*P*>0.05), as shown in Table-I. LRH was associated with significantly shorter surgery duration, lower time to anal exhaust, and shorter hospitalization time. As shown in Table-II, the intraoperative blood loss was considerably lower, while the number of lymph node dissections was greater than that of the ARH group (*P*<0.05).

**Table-I:** Comparison of Basic Clinical Characteristics between Two Groups.

Characteristics	LRH group (n=56)	ARH group (n=59)	t/χ^2^	P
Age (years), mean±SD	48.5±7.12	49.88±7.77	-0.992	0.323
BMI (kg/m^2^), mean±SD	23.34±2.95	23.50±2.52	-0.325	0.746
***Pathological type, n (%)***				
Squamous cell carcinoma	41 (73.21)	45 (76.27)	0.142	0.706
Adenocarcinoma	15 (26.79)	14 (23.73)		
***FIGO staging, n (%)***				
Ia	10 (17.86)	7 (11.86)	1.527	0.466
Ib	30 (53.57)	38 (64.41)		
IIa	16 (28.57)	14 (23.73)		
***Marital status, n (%)***				
Married	40 (71.43)	50 (84.75)	2.995	0.084
Unmarried/divorced	16 (28.57)	9 (15.25)		

***Note:*** ARH - abdominal radical hysterectomy; LRH - laparoscopic radical hysterectomy; BMI - body mass index; FIGO – International Federation of Gynecology and Obstetrics.

**Table-II:** Comparison of perioperative conditions between two groups.

Item	LRH group (n=56)	ARH group (n=59)	t/χ^2^	P
Surgical duration (minute), mean±SD	195.45±30.93	210.69±40.03	-2.292	0.024*
Intraoperative blood loss (ml), mean±SD	226.16±42.98	253.98±49.95	-3.194	0.002*
Duration of anal exhaust (day), M(P25/P75)	2 (2-3)	3 (2-4)	-3.228	0.001*
Number of lymph node dissections (piece), mean±SD	26.68±4.86	22.88±3.75	4.707	<0.001*
Duration of hospitalization (day), M(P25/P75)	6 (6-7)	10 (9-11)	-5.837	<0.001*

***Note:*** ARH - abdominal radical hysterectomy; LRH - laparoscopic radical hysterectomy; SD - standard deviation.

* P < 0.05, statistically significant.

**Fig.1:**
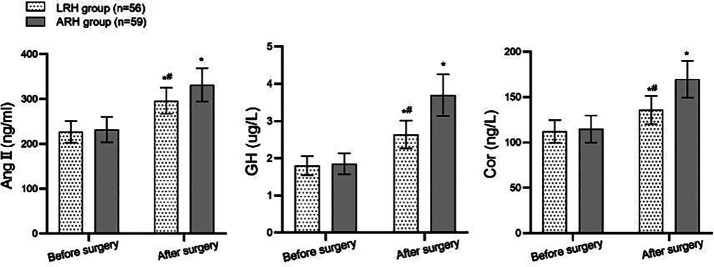
Comparison of two sets of stress response indicators. Compared with the same group before surgery, ^*^P<0.05; Compared with the ARH group, ^#^P<0.05; ARH - abdominal radical hysterectomy; LRH - laparoscopic radical hysterectomy; Ang II - angiotensin II; GH - growth hormone; Cor - cortisol.

**Fig.2:**
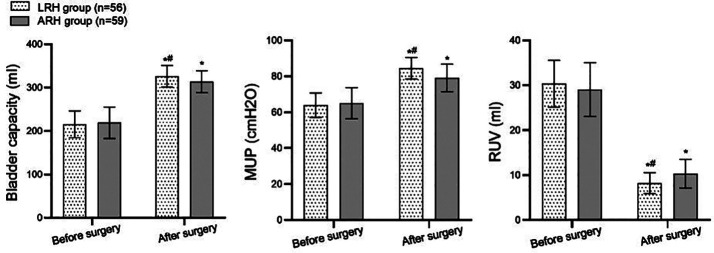
Compares the urodynamic states of the two groups. Compared with the same group before surgery, ^*^P<0.05; Compared with the ARH group, ^#^P<0.05; ARH - abdominal radical hysterectomy; LRH - laparoscopic radical hysterectomy; MUP - maximum urethral pressure; RUV - residual urine volume.

Before the surgery, there was no significant difference in the serum Ang II, GH and Cor levels between the two groups (*P*>0.05). These indices increased significantly after the surgery in both groups, but were substantially lower in the LRH group compared to the ARH group (P<0.05; Fig.1). Both groups’ preoperative bladder capacity, MUP, and RUV levels were comparable (*P*>0.05). After the surgery, the bladder capacity and MUP levels in both groups increased significantly and were considerably higher in the LRH group compared to the ARH group. Conversely, postoperative RUV decreased significantly compared to preoperative levels and was markedly lower in the LRH group than in the ARH group (P<0.05; Fig.2). The incidence of complications was significantly lower in the LRH group (3.57%) compared to the ARH group (16.95%) (*P*<0.05; Table-III).

**Table-III:** Comparison of incidence of complications between two groups.

Group	n	Intestinal obstruction	Urinary retention	Infection	Tubal injury	Total incidence rate
LRH group	56	0 (0.00)	1 (1.79)	1 (1.79)	0 (0.00)	2 (3.57)
ARH group	59	1 (1.69)	3 (5.08)	4 (6.78)	2 (3.39)	10 (16.95)
*χ^2^*						4.163
*P*						0.041*

***Note:***

* P < 0.05, statistically significant.

## DISCUSSION

This retrospective study analyzed clinical records of 115 patients with early-stage cervical cancer who underwent surgical treatment. The results showed that LRH was associated with a significantly shorter operation time, anal exhaust duration, and hospitalization length, as well as lower intraoperative blood loss and a higher number of dissected lymph nodes.

The results of this study are consistent with previous reports by Wang Y et al.,^14^ and Corrado G et al.^15^ and suggest that the benefits of LRH may be due to several factors. A shorter surgical duration reduces patient risks during the surgery and alleviates the burden on medical staff. The shortened duration of anal exhaust indicates a more rapid recovery of gastrointestinal function, which allows early and adequate nutrient intake and promotes overall physical recovery. At the same time, shorter hospitalization reduces the economic burden on patients and improves the efficiency of medical resource utilization. Reduced intraoperative blood loss in patients undergoing LRH lowers the risk of potential complications caused by excessive blood loss in patients, and a larger number of dissected lymph nodes ensures better tumor clearance, thereby improving the prognosis of patients.^16^-^18^

This study showed that while both surgical methods resulted in elevated levels of stress hormones AngII, GH and Cor, this stress response was significantly lower in the LRH group compared to patients who underwent ARH. Stress responses, induced by surgical trauma and severe pain, are known to suppress immune function, which can delay recovery and increase postoperative complications.^19^ In contrast, LRH is associated with reduced surgical trauma, improved visual field, and more precise lymph node dissection, which allows faster recovery and a lower incidence of stress-induced adverse effects.^5^,^6^,^20^

In terms of urodynamic indicators, LRH was associated with a significantly higher postoperative bladder capacity and MUP levels compared to ARH. Conversely, the postoperative RUV level in the LRH group was considerably lower than in the ARH group. Urodynamic examination of CC patients is conducive to the early prevention and treatment of acute urinary retention caused by bladder outlet obstruction, the early recovery of bladder function, and the reduction of urinary tract function damage in patients.^21^-^23^ The results of this study indicate that LRH can more effectively improve the urinary function of patients and reduce the occurrence of symptoms such as frequent urination, urgency, and incontinence, improving the patient’s quality of life.^21^ In agreement with previous reports, this study further confirms the advantages of LRH in protecting the urinary system of CC patients.^21^,^22^ However, previous studies have suggested that long-term changes in urodynamics may require longer follow-up to validate.^22^,^23^ Further studies with longer follow-ups are needed to confirm the conclusions of this analysis.

This study showed that the incidence of complications in the LRH group (3.57%) was significantly lower than in the ARH group (16.95%). These results suggest that LRH has higher safety. It is widely believed that due to its minimally invasive nature, less tissue damage, and more precise operation, LRH can reduce the risk of complications.^6^,^18^,^20^ While our results are consistent with the previous reports, it is important to note that the specific types and incidence of complications may vary depending on the inclusion criteria and follow-up time of different studies.^24^,^25^

Previous studies have indicated that minimally invasive surgery for CC patients is associated with higher recurrence and mortality rates compared to ARH.^2^,^13^ Moreover, LRH is associated with higher costs and requires high technical skills.^24^ Due to the lack of follow-up data in this study, only the perioperative treatment records of the two groups of patients were analyzed. Therefore, this study is one of the few that have analyzed stress levels and urodynamic indicators, confirming that LRH is more beneficial than ARH in the surgical treatment of early CC.

The present study offers valuable insight into the short-term physiological and functional outcomes associated with two widely used surgical techniques for early-stage cervical cancer. By simultaneously assessing perioperative metrics, serum stress biomarkers, and objective urodynamic parameters, the study extends beyond conventional surgical outcome evaluations, contributing to a more nuanced understanding of early postoperative recovery. The use of routinely collected clinical data from actual practice settings also enhances the practical relevance of the findings. Despite its contributions, several aspects warrant further investigation. Prospective studies with longer follow-up durations are needed to evaluate long-term oncologic efficacy, including recurrence and survival outcomes. Future research should also address quality-of-life metrics, the potential impact of nerve-sparing techniques, and the influence of surgical experience on functional recovery, particularly with respect to bladder and sexual function.

### Limitations:

As a retrospective, single-center analysis, it is subject to inherent selection bias and limited generalizability. The sample size was relatively small and determined by the availability of eligible cases during the study period, without a priori sample size calculation or power analysis. These factors may reduce the statistical power of some comparisons. Moreover, the inclusion of only patients with complete clinical data may have introduced additional bias. The surgical outcomes could also have been influenced by technical variability, including differences in surgeons’ experience. Finally, the study focused solely on short-term perioperative indicators, stress biomarkers, and urodynamic changes. Longer follow-up studies are necessary to assess long-term oncologic outcomes, including recurrence, survival, and patient-reported quality of life.

## CONCLUSION

Compared with the ARH approach, the LRH for treating early CC is associated with higher benefits in the early postoperative period, including reduced surgical trauma, alleviated stress response caused by invasive surgical procedures, improved urodynamic status, a reduced risk of complications, and shorter postoperative recovery.

### Authors’ contributions:

**PG:** Study design, literature search, manuscript writing, revision, validation and is responsible for the integrity of the study.

**HW and YW:** Data collection, data analysis and interpretation. Critical Review.

All authors have read and approved the final manuscript.
